# Comprehensive analysis of 84 *Faecalibacterium prausnitzii* strains uncovers their genetic diversity, functional characteristics, and potential risks

**DOI:** 10.3389/fcimb.2022.919701

**Published:** 2023-01-04

**Authors:** Zipeng Bai, Na Zhang, Yu Jin, Long Chen, Yujie Mao, Lingna Sun, Feifei Fang, Ying Liu, Maozhen Han, Gangping Li

**Affiliations:** ^1^ School of Life Sciences, Anhui Medical University, Hefei, Anhui, China; ^2^ Division of Gastroenterology, Union Hospital, Tongji Medical College, Huazhong University of Science and Technology, Wuhan, China

**Keywords:** *Faecalibacterium prausnitzii*, probiotics, fatty acid metabolism, probiotic potential risk, probiotic potential risk assessment indices

## Abstract

*Faecalibacterium prausnitzii* is a beneficial human gut microbe and a candidate for next-generation probiotics. With probiotics now being used in clinical treatments, concerns about their safety and side effects need to be considered. Therefore, it is essential to obtain a comprehensive understanding of the genetic diversity, functional characteristics, and potential risks of different *F. prausnitzii* strains. In this study, we collected the genetic information of 84 *F . prausnitzii* strains to conduct a pan-genome analysis with multiple perspectives. Based on single-copy genes and the sequences of 16S rRNA and the compositions of the pan-genome, different phylogenetic analyses of *F. prausnitzii* strains were performed, which showed the genetic diversity among them. Among the proteins of the pan-genome, we found that the accessory clusters made a greater contribution to the primary genetic functions of *F. prausnitzii* strains than the core and specific clusters. The functional annotations of *F. prausnitzii* showed that only a very small number of proteins were related to human diseases and there were no secondary metabolic gene clusters encoding harmful products. At the same time, complete fatty acid metabolism was detected in *F. prausnitzii*. In addition, we detected harmful elements, including antibiotic resistance genes, virulence factors, and pathogenic genes, and proposed the probiotic potential risk index (PPRI) and probiotic potential risk score (PPRS) to classify these 84 strains into low-, medium-, and high-risk groups. Finally, 15 strains were identified as low-risk strains and prioritized for clinical application. Undoubtedly, our results provide a comprehensive understanding and insight into *F. prausnitzii*, and PPRI and PPRS can be applied to evaluate the potential risks of probiotics in general and to guide the application of probiotics in clinical application.

## Introduction

*Faecalibacterium prausnitzii* is a gram-negative and strictly anaerobic rod-shaped bacterium that is one of the most abundant commensal bacterial species in the colons of healthy humans (Duncan et al., 2002; Hold et al., 2003). *F. prausnitzii* was initially classed as a member of the *Fusobacterium* genus of the Firmicutes phylum. However, guided by phylogenetic analysis based on 16S rRNA sequencing, *F. prausnitzii* was identified as a new genus and can be classified into two phylogroups and three clusters (Duncan et al., 2002; Lopez-Siles et al., 2012; Benevides et al., 2017; Lopez-Siles et al., 2017). The importance of this commensal bacterium as a component of the healthy human microbiota has been highlighted by an increasing number of studies (Passera et al., 2016; Martín et al., 2017).

*F. prausnitzii* is well known as one of the most abundant butyrate-producing bacteria in the gastrointestinal tract (Flint et al., 2014). Butyrate plays a major role in gut physiology, and it has pleiotropic effects in the intestinal cell life cycle and numerous beneficial effects for health through protection against pathogen invasion, modulation of the immune system, and reduction of cancer progression (Macfarlane and Macfarlane, 2011). The metabolic activity of *F. prausnitzii* is not restricted to the production of butyrate; it can also consume acetate and produce carbon dioxide, formate, and D-lactate. Additionally, *F. prausnitzii* can hydrolyze fructose, oligosaccharides, pectin, starch, and even inulin (Lopez-Siles et al., 2012). The high metabolic activity of *F. prausnitzii* is beneficial not only for colonocytes but also for host metabolism. *F. prausnitzii*, as a commensal bacterium, has an anti-inflammatory property that has been identified based on human clinical data (Miquel et al., 2015). This protective mechanism presumably involves the inhibition of proinflammatory cytokines, such as nuclear factor-κB, and has a key role in the stimulation of anti-inflammatory cytokine secretion (such as IL-10) by active molecules (Zhang et al., 2014). Changes in the abundance of *F. prausnitzii* have been linked to dysbiosis not only in gastrointestinal disorders but also in metabolic syndromes and Alzheimer’s disease (Ueda et al., 2021). Investigations have revealed that the abundance of *F. prausnitzii* is low in both ulcerative colitis (UC) and Crohn’s disease (CD) patients compared with healthy people (Varela et al., 2013; Radhakrishnan et al., 2022). A reduction in *Faecalibacterium* spp. in fecal samples has been reported to be associated with an increase in irritable bowel syndrome (IBS) symptoms (Miquel et al., 2016). A decrease in *Faecalibacterium* spp. has been observed in colorectal cancer patients in comparison with healthy controls (Wu et al., 2013). Unlike the changes in gastrointestinal disorders, there were inconsistent trends in metabolic syndromes. For example, a previous study reported that the relative abundance of *F. prausnitzii* is increased in obese patients in contrast to healthy individuals (Furet et al., 2010). Lower abundances of *Faecalibacterium* were reported in patients with type 2 diabetes and non-alcoholic fatty liver disease (Qin et al., 2012; Zhu et al., 2013). There was a higher abundance of proinflammatory bacteria (e.g., *Escherichia/Shigella*) and a lower distribution of anti-inflammatory bacteria (e.g., *Bacillus fragilis, F. prausnitzii*) in amyloid-positive Alzheimer’s disease patients than in healthy subjects (Lee et al., 2018). It can be concluded that the population level and functions of *F. prausnitzii* are associated with host health. However, their causes and consequences are not clearly understood, and much information is lacking regarding which subspecies of *F. prausnitzii* are important under which conditions.

Given the important role of *F. prausnitzii* in human health and the fact that there have been no previous reports of its pathogenic characteristics, there is a clear potential for this species as a next-generation probiotic. Despite its importance in human health, only a few microbiological studies have been performed to isolate novel *F. prausnitzii* strains to better understand the biodiversity and phylogenetic diversity of this beneficial commensal species. With regard to the phylogenetic relationships of *F. prausnitzii* strains, several previous studies have found that they can be classified into two phylogroups and three clusters according to their 16S rRNA sequences, and the results support their membership of two different genomospecies or genomovars (Duncan et al., 2002; Lopez-Siles et al., 2012; Benevides et al., 2017; Lopez-Siles et al., 2017). Differences in enzyme production, antibiotic resistance, and immunomodulatory properties were found to be strain dependent (Martín et al., 2017). As of 15 July 2021, the genomes of 84 *F . prausnitzii* strains have been sequenced; six strains have been completely sequenced, and the rest have been mainly assembled at the contig and scaffold levels (Bag et al., 2017). Moreover, the genetic diversity and functional traits of *F. prausnitzii*, especially the CRISPR/Cas system, which is widely used in editing the genetic elements of microbiota (Horvath and Barrangou, 2010), and the compositions of virulence factors (VFs), antibiotic resistance genes (ARGs), and pathogenic genes (PGs), remain unclear. Therefore, a comprehensive analysis of the characteristics of *F. prausnitzii* is urgently needed to support to its clinical application.

In this study, we collected the available genomic datasets of 84 *F . prausnitzii* strains and conducted a comparative analysis to investigate the strains’ genetic diversity and functional traits, including cluster of orthologous group (COG) analysis, VFDB database annotation, CARD database annotation, PHI database annotation, KEGG database annotation, metabolic pathways analysis, and CRISPR/Cas composition analysis. Importantly, we proposed two indices, namely, the probiotic potential risk index (PPRI) and probiotic potential risk score (PPRS), based on the profiles of ARGs, VFs, and PGs, to estimate the potential risks of the clinical application of 84 *F . prausnitzii* strains, and these indices can be applied to other suitable probiotics.

## Material and methods

### Collection of genomic datasets of *F. prausnitzii*

To obtain the available genetic information of *F. prausnitzii*, we used *F. prausnitzii* as a keyword and searched the genome/RefSeq databases of NCBI on 15 July 2021. As a result, we collected the genome and protein sequences of 84 *F . prausnitzii* strains and downloaded them from the RefSeq database of NCBI for comparative genomic analysis. The assembly levels, genome assembly lengths, GC content (%), the numbers of proteins or genes, and the sources of these strains were obtained from NCBI and recorded in **Supplementary Table 1**.

### Identification of protein orthologs of *F. prausnitzii*

To obtain an in-depth understanding of the pan-genome of *F. prausnitzii*, all proteins derived from 84 *F . prausnitzii* strains were used to construct protein families using OrthoMCL (version: 2.0.9) (Li et al., 2003) with BLAST. The E-value threshold was set to 1e-5 and the inflation parameter threshold was set to 1.5 (Zhong et al., 2018). Subsequently, a total of 8,420 homologous clusters were generated. To better understand the functional compositions of *F. prausnitzii*, these homologous clusters were divided into three categories, namely, core (representing the proteins shared by 84 *F . prausnitzii* strains), specific (representing the proteins detected only in one *F. prausnitzii* strain), and accessory groups (the rest of the proteins shared by at least two strains but not by all). Finally, 375 and 303 homologous clusters were classified into core groups and single-copy gene families, respectively. Additionally, detailed information about the reconstructed pan-genome of *F. prausnitzii* was obtained. Each identified orthogroup protein, including their original *F. prausnitzii* strain, accession number, protein sequence, and cluster number in the 84 *F. prausnitzii* strains, can be downloaded from the website: https://github.com/Basspoom/Detailed-information-on-the-reconstructed-pangenome-of-Faecalibacterium-prausnitzii.git.

### Construction of phylogenetic trees for *F. prausnitzii*

To determine the evolutionary relationships of the 84 *F . prausnitzii* strains, three types of phylogenetic tree were constructed using different strategies. First, 16S rRNA sequences of the 84 *F . prausnitzii* strains were used to generate a phylogenetic tree with the maximum likelihood method. Then, the protein sequences for 303 single-copy gene families were aligned using MUSCLE (version 3.8.31) (Edgar, 2004) with default parameters, and then these alignment results were concatenated and used as an import sequence document with MEGA-X (version 10.2.5) (Kumar et al., 2018) to construct the phylogenetic tree of 84 *F . prausnitzii* strains using the neighbor-joining (NJ) method (Saitou and Nei, 1987). Additionally, the Manhattan distance was calculated based on the pan-genome composition of *F. prausnitzii*, which was used to generate a phylogenetic tree with the unweighted pair group average method (UPGMA).

### Functional annotations of *F. prausnitzii*

To obtain an in-depth understanding of the functional compositions of *F. prausnitzii*, the proteins of homologous clusters were annotated against the COG and CAZyme databases. First, the representative protein sequences of 8,240 homologous clusters were annotated with the COG database (https://www.ncbi.nlm.nih.gov/COG/) (Galperin et al., 2019) using BLASTP with the following parameters: the E-value threshold was set to 1e-5 and the top annotation result was selected as the best functional trait for each homologous cluster and assigned to one of 25 functional categories (Han et al., 2020). Second, these representative protein sequences were annotated against the CAZyme database (Lombard et al., 2014), downloaded from dbCAN (Huang et al., 2018), using hmmscan of HMMER (version 3.1b1) (Makela et al., 2018) with default settings. The annotated results were summarized as glycosyltransferase (GT), glycoside hydrolase (GH), carbohydrate esterase (CE), carbohydrate-binding module (CBM), auxiliary activity (AA), polysaccharide lyase (PL), and S-layer homology (SLH).

### Detection of secondary metabolism gene clusters and pathway construction in *F. prausnitzii*

KOALA (KEGG Orthology And Links Annotation) (Kanehisa et al., 2017) and AntiSMASH 6.0 (Medema et al., 2011) were used to gain more insight into the metabolic pathways, in particular, the secondary metabolism gene clusters of *F. prausnitzii*. Specifically, the proteins of each *F. prausnitzii* strain were annotated with the KEGG database using two online tools, namely, GhostKOALA and KofamKOALA under KOALA, to obtain detailed annotation results (Kanehisa et al., 2016b). Furthermore, EnrichM (version 0.64) (Liu et al., 2021) was used to obtain the KO matrix of *F. prausnitzii* to construct the metabolic pathways, such as the pathway of the metabolism of fatty acids of *F. prausnitzii*, which was constructed using the reconstruction function of KEGG Mapper (https://www.genome.jp/kegg/mapper/), a program that provides an in-depth understanding of the synthesis of fatty acids. In addition, AntiSMASH (https://antismash.secondarymetabolites.org/) was used to detect the potential secondary metabolite biosynthesis gene clusters of the 84 *F. prausnitzii* strains with the following parameters: detection strictness and relaxed to identify well-defined clusters (Blin et al., 2021).

### Prediction of the CRISPR/Cas system of *F. prausnitzii*

To explore the profiles of the CRISPR/Cas system and obtain a better genetic background of *F. prausnitzii* strains, we investigated the components of the CRISPR/Cas system, including CRISPR arrays and Cas genes, using the online tool CrisprCasFinder (https://crisprcas.i2bc.paris-saclay.fr/) (Couvin et al., 2018). In particular, the size of the flanking region of each analyzed CRISPR array was set to 100, and the remaining parameters were set to default values. Furthermore, 10 strains that contained more sequences than CrisprCasFinder could deal with were analyzed using the CRISRPCasMeta tool with default settings, namely, *F. prausnitzii* KLE1255, *F. prausnitzii* 2789STDY5834930, *F. prausnitzii* CNCM | 4542, *F. prausnitzii* CNCM | 4546, *F. prausnitzii* BIOML-B15, *F. prausnitzii* BIOML-B16, *F. prausnitzii* BIOML-B6, *F. prausnitzii* BIOML-B1, *F. prausnitzii* SSTS Bg7063, and *F. prausnitzii* JG BgPS064.

### Profiles of virulence factors, antibiotic resistance genes, and pathogenic genes of *F. prausnitzii*

To obtain an in-depth understanding of the genetic background of *F. prausnitzii*, the composition of virulence factors (VFs), antibiotic resistance genes (ARGs), and pathogenic genes (PGs) of *F. prausnitzii* were profiled. In brief, the setA database of the pathogenic Virulence Factor Database (VFDB) was downloaded from the official website (http://www.mgc.ac.cn/VFs/) (Chen et al., 2005). Then, the setA database was locally distributed and used for protein annotations of *F. prausnitzii* with DIAMOND (version: 0.8.36) (Buchfink et al., 2021). The annotation results were screened with a similarity of ≥80% and a coverage of ≥70% to obtain a more accurate annotation of virulence factors (Han et al., 2022). Then, eight kinds of virulence factor were filtered. Second, a famous ARG database, the Comprehensive Antibiotic Resistance Database (CARD) (McArthur et al., 2013), was downloaded (https://card.mcmaster.ca/) and used to detect potential ARGs from 8,240 homologous protein clusters with the default parameters; then, the results were filtered using a similarity of ≥80% and a coverage of ≥70% (Han et al., 2022). Third, to obtain the pathogenicity of *F. prausnitzii*, the Pathogen Host Interactions database (PHI-base, http://www.phi-base.org/) (Urban et al., 2020), a web-accessible database that focuses on pathogenicity, virulence, and effector genes from different pathogens, was applied to identify potential pathogenic genes. The results were filtered using the following parameters: similarity of ≥80% and coverage of ≥70%.

### Estimation of the potential risks of *F. prausnitzii* strains

Previous studies have demonstrated that the beneficial human gut microbe *F. prausnitzii* is a candidate ‘probiotic of the future’ or ‘next-generation probiotic’ because it can produce high amounts of butyrate and several anti-inflammatory compounds (Khan et al., 2014; He et al., 2021; Maioli et al., 2021). In recent years, increasing applications of probiotics for the clinical treatment of diseases have been reported. Nevertheless, dangerous problems caused by probiotics occasionally occur, such as the adverse effects of the probiotic *Lactobacilli precautions* (Castro-Gonzalez et al., 2019) in recent case reports, including bacteremia and/or sepsis (Passera et al., 2016), abscesses (Pararajasingam and Uwagwu, 2017), and endocarditis (Boumis et al., 2018), suggesting that the safety of probiotics should be considered. However, the safety issues of probiotics have not been directly addressed, and the potential risk of probiotics has not been evaluated. Hence, to evaluate the potential risk of *F. prausnitzii* strains, we proposed two indices, namely, the PPRI and PPRS, based on the composition of ARGs, VFs, and PGs, which can provide guidance for choosing probiotics for the clinical treatment of diseases. PPRI was designed as a one-dimensional combination vector with three variables with the following equation:


$$PPRI=c\;\left(N_{ARGs},N_{VFs},N_{PGs}\right),$$

where *N*_ARGs_ represents the number of ARGs of a strain, *N*_VFs_ represents the number of VFs of a strain, and *N*_PGs_ represents the number of PGs of a strain. To intuitively assess the potential risk of probiotic strains, including *F. prausnitzii* strains, we designed a PPRS that calculated the Euclidean distance of *F. prausnitzii* strains based on the PPRI with the following equation:


$$\text{PPRS}=\sqrt{N_{ARGs}{\;}^{2}+N_{VFs}{\;}^{2}+N_{PGs}{\;}^{2}}$$

## Results and discussion

### Pan-genome construction and analysis

In the present study, to conduct a comparative analysis of *F. prausnitzii*, 84 available genome sequences, including complete and draft genomes, as well as their corresponding protein sequences for *F. prausnitzii* strains, were collected and downloaded from NCBI. The detailed information of these 84 *F . prausnitzii* strains is summarized in **Supplementary Table 1**, including the levels of assembly, the sizes of the genomes, the counts of genes and proteins, and their separation sources. With regard to the sources of these 84 *F . prausnitzii* strains, we found that a majority of strains were isolated from the feces of humans, such as *F. prausnitzii* KLE1255, *F. prausnitzii* APC918/95b, and *F. prausnitzii* A2165, which was consistent with the finding that *F. prausnitzii* is dominant in the intestinal tract of humans (Miquel et al., 2013). Although the genomes of 84 *F . prausnitzii* strains have been sequenced, only six strains have been completely sequenced, and the rest of the strains have been mainly assembled at the contig and scaffold levels. The number of scaffolds and contigs ranged from 1 to 1,135, and the sizes of the assembled genomes of *F. prausnitzii* strains ranged from 2.66 to 3.42 Mb. The GC content (%) ranged from 52.9% to 58.1%, and the numbers of proteins ranged from 1,991 to 3,264 (**Supplementary Table 1**).

**Figure 1:**
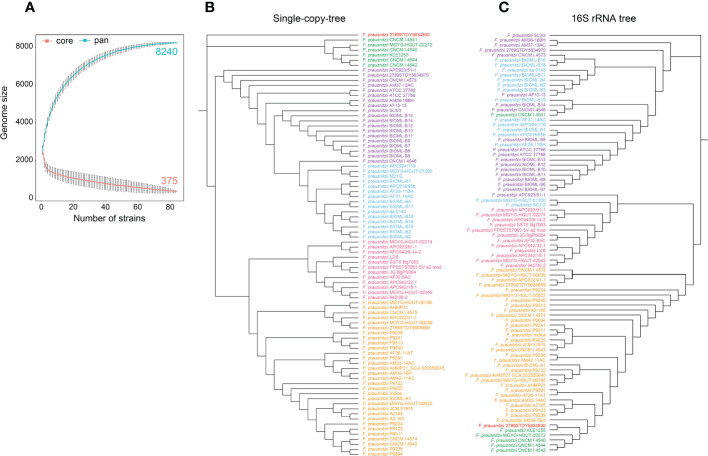
Pan-genome structure and phylogenetic analysis of *F. prausnitzii*. **(A)** Pan-genome structure of *F. prausnitzii*, showing increases and decreases in gene families in the core genome (blue) and pan-genome (red). The core geome of *F. prausnitzii* showed an open trend compared with its pan-genome. **(B)** A neighbor-joining phylogenetic tree was constructed based on the protein sequences for 303 single-copy gene families of these 84 *F. prausnitzii* strains, and they could be divided into six groups according to their evolutionary relationships. **(C)** A maximum likelihood phylogenetic tree was constructed using the 16S rRNA sequences of the 84 *F. prausnitzii* strains. Strains of the same name were marked with the same color in **(B, C)**.

To characterize the differences in genomic features among *F. prausnitzii* strains, we generated protein orthologs based on 194,877 high-quality proteins from 84 *F . prausnitzii* strains, and a total of 8,240 homologous clusters were identified. In addition, 375 homologous clusters were detected in 84 *F . prausnitzii* strains (**Figure 1A**), which were defined as the core genomes. In particular, 303 homologous clusters of core genomes were identified as single-copy core protein families. Additionally, 207 homologous clusters that were detected in only one strain of the 84 *F . prausnitzii* strains were classified into specific genes (unique genes), and the number of specific genes ranged from 1 to 149 in these 84 *F . prausnitzii* strains. Specifically, the number of specific genes in most *F. prausnitzii* strains was between 1 and 12, except for *F. prausnitzii* 2789STDY5834930, which contained 149 unique genes. In addition, the number of accessory families of *F. prausnitzii* strains ranged from 739 to 2,609 (**Supplementary Table 2**).

The core genome of *F. prausnitzii* showed an open trend compared with its pan-genome (**Figure 1A**). With the increase in the number of strains, the genome size of the core genes gradually decreased; finally, 375 core genes were identified. In addition, we found that only 4.5% of the pan-genome remained unchanged, while the remaining 95.5% was variable between strains, indicating that the pan-genome showed a high degree of genomic variability (**Figure 1A**). These results revealed that the genetic elements and functional traits of 84 *F . prausnitzii* strains were varied and contributed to the adaptation of *F. prausnitzii* strains to the intestinal niche.

### Phylogenetic analysis of *F. prausnitzii* strains

To compare the similarity and genetic distance of the 84 *F. prausnitzii* strains, three types of phylogenetic trees were constructed based on the protein sequences for 303 single-copy gene families, their 16S rRNA sequences (**Figures 1B, C**), and the pan-genome of the 84 *F . prausnitzii* strains. Specifically, the protein sequences for 303 single-copy gene families were aligned, and then these alignment results were concatenated and used to construct the phylogenetic tree of 84 *F . prausnitzii* strains using the NJ method (**Figure 1B**). In addition, based on the sequences of 16S rRNA, we used MEGA-X to construct a phylogenetic tree using the maximum likelihood method (**Figure 1C**). As shown in **Figures 1B, C**, we found that these 84 *F. prausnitzii* strains could be divided into six groups according to their evolutionary relationships based on the two different construction strategies. Importantly, we compared these two phylogenetic trees with those of *F. prausnitzii* from studies by Fitzgerald et al. (2018) and Benevides et al. (2017). Fitzgerald et al. (2018) constructed a maximum likelihood phylogenetic tree of the family *Ruminococcaceae* based on concatenated alignments of 245 highly conserved proteins. In their study, *F. prausnitzii* could be divided into three groups, namely, I, IIa, and IIb. Compared with our single-copy tree (**Figure 1B**), group I corresponds to our blue and purple groups, group IIa corresponds to our green group, and group IIb corresponds to our pink and yellow groups. Benevides et al. (2017) also grouped *Faecalibacterium* into three different groups using 16S rRNA sequence clustering. As more *F. prausnitzii* strains were isolated and sequenced, more genomes were used in the present study and more detailed classification results were obtained. These results suggest that the evolutionary relationships of *F. prausnitzii* strains are complex, indicating the need for a pan-genomic analysis of *F. prausnitzii*.

Meanwhile, there were great discrepancies in the composition of the phylogenetic trees constructed using the two different strategies (**Figures 1B, C**). With regard to *F. prausnitzii* 2789STDY5834930, we found that it was separately grouped in the single-copy tree, revealing that it has the longest evolutionary time and may be the oldest species in the genus *Faecalibacterium*. Additionally, based on the composition of the pan-genome of the 84 *F. prausnitzii* strains, the UPGMA method was used to construct a phylogenetic tree to reveal the relationships between the *F. prausnitzii* strains (**Supplementary Figure 1**). The UPGMA tree was also significantly different from the phylogenetic trees constructed using the previous two strategies.

Overall, these results showed that the genetic elements and the evolutionary relationships of the *F. prausnitzii* strains were varied and complex, suggesting that a pan-genome analysis is essential to obtain an in-depth understanding of *F. prausnitzii* that benefits its applications as a new probiotic. Hence, we evaluated the functional compositions of *F. prausnitzii* strains from different perspectives, including COG annotations, CAZyme, metabolic pathways, secondary metabolism (in particular, fatty acid metabolism), the composition of CRISPR/Cas systems, and the composition of ARGs, VFs, and PGs, and constructed two indices, namely, the PPRI and PPRS, to evaluate the potential risk of *F. prausnitzii* strains and provide guidance for their application.

### COG annotations for *F. prausnitzii* strains

**Figure 2:**
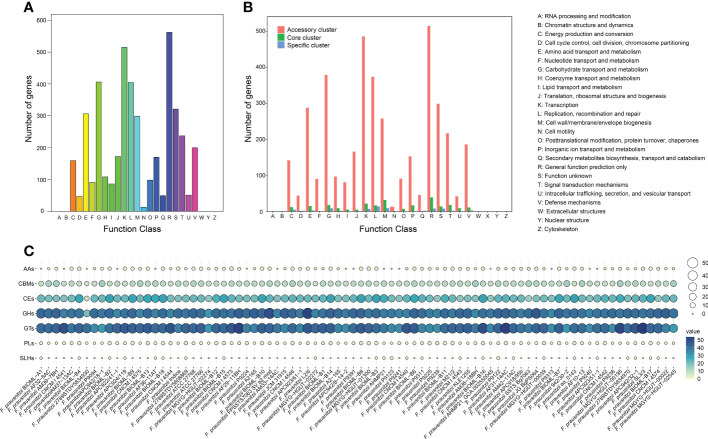
Pan-genome function of *F. prausnitzii*. **(A)** COG function classification of expressed contig sequences. **(B)** Distribution of COG categories in *F. prausnitzii* accessory, core, and unique genomes. The numbers of genes assigned by COG categories in the accessory cluster (red bars), core cluster (green bars), and unique cluster (blue bars) are shown. The functions of these genes are summarized on the right. **(C)** Distribution of CAZymes of pan-genome in 84 *F. prausnitzii* strains. The CAZymes classified from the pan-genome clusters were mainly divided into carbohydrate esterase (CE), glycoside hydrolase (GH), and glycosyltransferase (GT).

To gain insight into the functional characteristics of *F. prausnitzii* strains, we annotated the 8,420 homologous clusters against the COG database (Galperin et al., 2019) (**Figure 2A**) and reorganized the functional traits based on the groups of accessory, core, and specific clusters (**Figure 2B**). As shown in **Figure 2A**, we found that several homologous clusters were annotated as the categories ‘only general function prediction’ (13.1%) and ‘function unknown’ (7.5%), which suggested that the functional traits of *F. prausnitzii* strains are unknown.

Subsequently, the annotations were divided into three groups according to their affiliation. Their numbers and proportions are presented in detail in **Supplementary Table 3**. We found that the functional traits of the core cluster contain diverse functions and play a basic role in the maintenance of primary cellular processes, including ‘DNA replication and transcription’, ‘cell signal transduction’, and ‘information storage and handling’ (**Figure 2B**). The functional annotations of unique genes were mainly annotated to ‘energy production and conversion’, ‘amino acid transport and metabolism’, ‘transcription’, and other similar categories (**Figure 2B**). We found that a proportion of unique genes (1.7%) were annotated. By contrast to core and unique clusters, a high proportion of the proteins (92.4%) classified into accessory clusters could be assigned to ‘transcription’, ‘replication, recombination, and repair’, ‘cell wall/membrane/envelope biogenesis’, ‘signal transduction mechanisms’, and the metabolism of biological macromolecules (**Figure 2B**). Moreover, the distributions of these accessory proteins in the 84 *F. prausnitzii* strains was varied. For example, the category ‘replication, recombination, and repair’ contained genes involved in mobile elements (transposase, recombinase, and integrase genes), indicating the presence of potential horizontal gene transfer events (Soucy et al., 2015). Overall, these results suggest that the composition of the accessory cluster contributes to the diversity of functional traits of different *F. prausnitzii* strains.

### Identification of CAZymes for *F. prausnitzii* strains

To obtain a comprehensive understanding of the catalytic abilities of carbohydrates and carbohydrate metabolism in *F. prausnitzii*, we annotated the 8,240 homologous clusters of the 84 *F . prausnitzii* strains against the CAZyme database and explored the distribution pattern of carbohydrate metabolism in these strains (**Figure 2C**; **Supplementary Tables 4** and **5**). We found that the functional traits associated with carbohydrate metabolism in the *F. prausnitzii* strains were mainly annotated as carbohydrate esterase (CE), glycoside hydrolase (GH), and glycosyltransferase (GT, **Figure 2C**). Specifically, in the present study, sixteen kinds of GTs were detected in the 84 *F . prausnitzii* strains (**Supplementary Figure 2**; **Supplementary Table 5**). In particular, GT2 and GT4 were predominant in *F. prausnitzii* (**Supplementary Figure 2**). In general, the primary function of GTs is associated with glycoside synthesis, and their substrates are diverse (Lairson et al., 2008). According to the description of GT2 and GT4 in a previous study, the GT2 and GT4 families mainly contain several glycosyltransferases, such as chitin synthase, cellulose synthase, and α-glucose transferase (Bohra et al., 2019). Additionally, a report by Martinez-Fleites et al. (2006) pointed out that WaaG in the GT4 family catalyzes a key step in lipopolysaccharide synthesis. Therefore, given the high proportions of GT2 and GT4 in the *F. prausnitzii* strains, we speculated that these strains have the potential metabolic ability of chitin, cellulose, and lipopolysaccharide.

Additionally, 10 kinds of CEs were detected in 84 *F. prausnitzii* strains (**Supplementary Figure 2**, **Supplementary Table 5**). CE1 and CE10 were present in slightly higher numbers in *F. prausnitzii*. The function of CEs is to participate in the metabolism of carbohydrate esters (Armendariz-Ruiz et al., 2018). Genes putatively coding for the CAZyme families of carbohydrate esterases CE1 and CE10 have been suggested to have lifestyle-specific gene expression patterns (de Vries and de Vries, 2020).

Moreover, 34 kinds of GHs were detected in 84 *F. prausnitzii* strains (**Supplementary Figure 2**, **Supplementary Table 5**). The content of each GH in *F. prausnitzii* was similar and low. The function of GHs is to carry out the hydrolysis reaction of glycosides (Janecek and Svensson, 2016).

### Construction of potential metabolic pathways for *F. prausnitzii* strains

**Figure 3:**
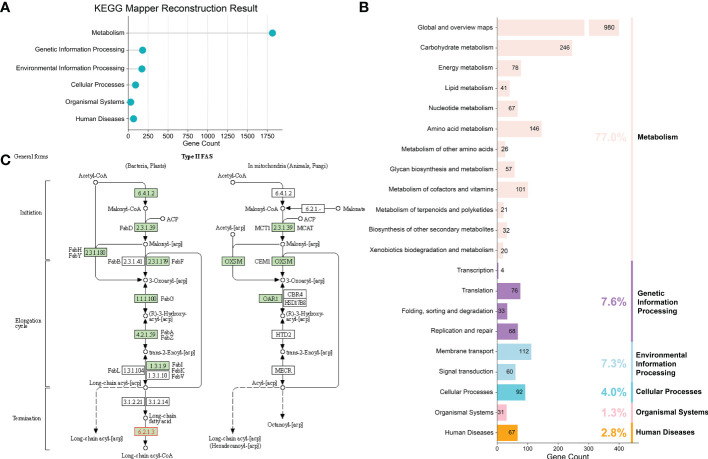
Construction of potential metabolic pathways for *F. prausnitzii* strains. **(A)** KEGG reconstruction of *F. prausnitzii*. The annotation results showed that the proteins of the homologous clusters of *F. prausnitzii* were mainly involved in ‘metabolic process’, ‘genetic information processing’, ‘environmental information processing’, ‘cellular processes’, ‘organismal systems’, and ‘human diseases’. **(B)** Detailed functional categories of KEGG reconstruction. **(C)** Fatty acid metabolism pathway of *F. prausnitzii* constructed *via* KEGG Mapper – Reconstruct Pathway. *F. prausnitzii* relies on the enzymes marked in green for fatty acid metabolism.

To obtain an in-depth understanding of the potential metabolic pathways of *F. prausnitzii*, we profiled the functional traits of *F. prausnitzii* strains with different strategies, including using the annotations obtained from KEGG databases (Kanehisa et al., 2016a) and secondary metabolic gene clusters predicted by AntiSMASH (Medema et al., 2011), as well as constructing the fatty acid metabolism pathway. First, we applied two tools, namely, GhostKOALA and KofamKOALA, to annotate the proteins of the 84 strains against the KEGG database (Kanehisa et al., 2016a; Kanehisa et al., 2017). Based on the annotations of these two tools for the 84 *F . prausnitzii* strains, the results are summarized in **Supplementary Tables 5**, **6**. Specifically, the annotation results showed that the proteins of the homologous clusters of *F. prausnitzii* were mainly involved in ‘metabolic process’ (77%), ‘genetic information processing’ (7.6%), ‘environmental information processing’ (7.3%), ‘cellular processes’ (4.0%), ‘organismal systems’ (1.3%), and ‘human diseases’ (2.8%, **Figures 3A, B**). Furthermore, among 8,240 homologous clusters of 84 *F . prausnitzii* strains, a total of 2,420 homologous clusters (29.4%) were annotated. These functional categories can be divided into ‘protein families: signaling and cellular process’, ‘protein families: genetic information process’, ‘environmental information processing’, and so on (**Figure 3B**). The details of these functional categories are provided in **Supplementary Table 7**.

**Figure 4:**
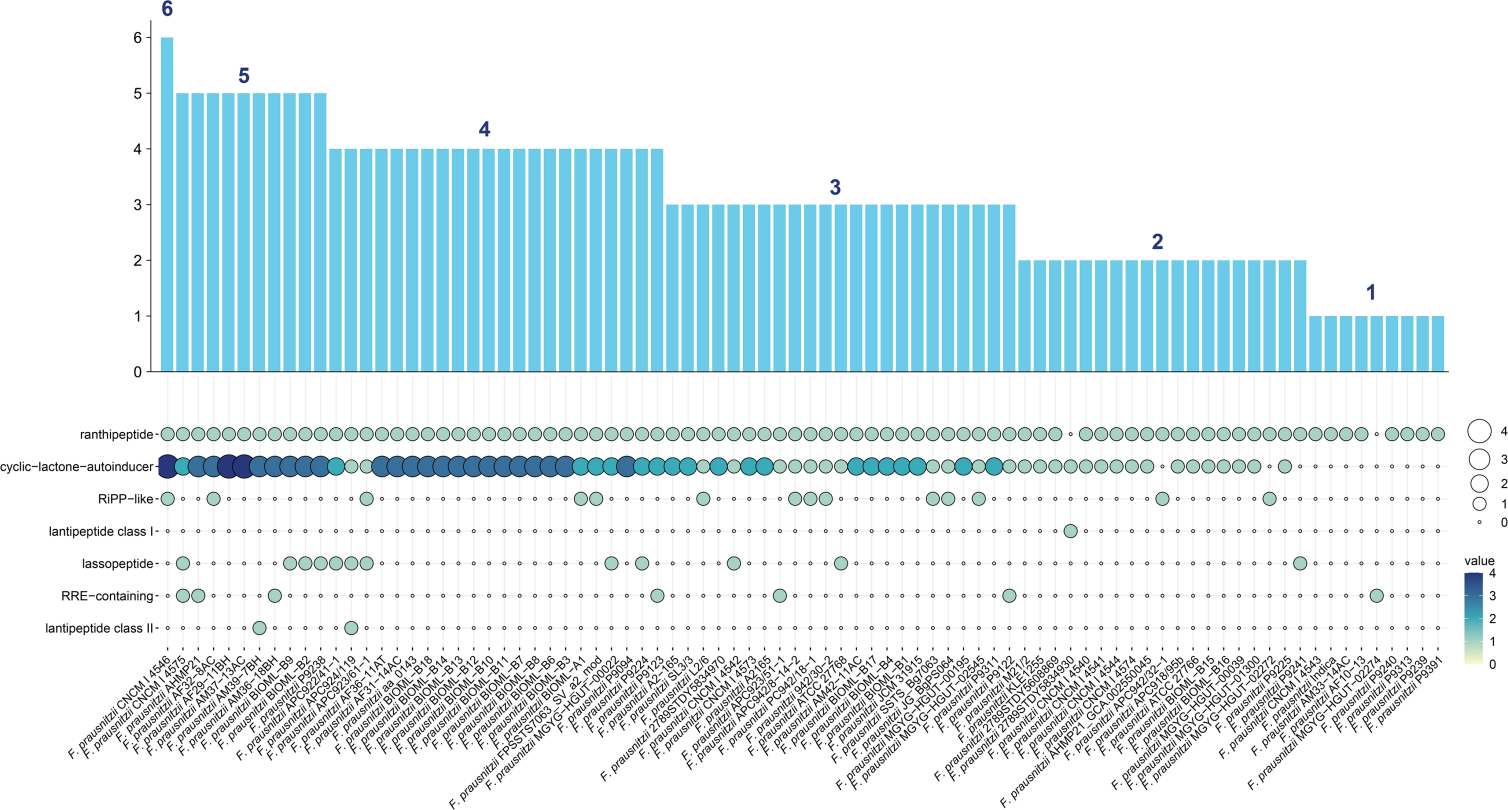
BGC carrying status of *F. prausnitzii*. Seven categories of secondary metabolism gene clusters (referred to as biosynthesis gene clusters, BGCs) from 84 F*. prausnitzii* strains were discovered: ranthipeptide, cyclic-lactone-autoinducer, RiPP-like, lantipeptide class I, lassopeptide, RRE-containing, and lantipeptide class II. The ordinate of the bar graph is the sum of the number of BGCs carried by each strain. Strains were arranged from high to low by the quantity of BGCs they carried.

Second, we used AntiSMASH (Medema et al., 2011) to detect the secondary metabolic gene clusters (referred to as biosynthesis gene clusters, BGCs) of *F. prausnitzii* strains (**Supplementary Table 8**). Seven categories of BGCs were detected from 84 *F . prausnitzii* strains (ranthipeptide, cyclic-lactone-autoinducer, RiPP-like, lantipeptide class I, lassopeptide, RRE-containing, and lantipeptide class II) (**Figure 4**). In particular, we found that a majority of *F. prausnitzii* strains (85.7%) harbor the ranthipeptide and cyclic-lactone-autoinducer gene clusters, but only a few strains (39.2%) contain RiPP-like, lantipeptide class I, lassopeptide, RRE-containing, and lantipeptide class II gene clusters (**Figure 4**).

Third, several previous studies have reported that the *F. prausnitzii* strain is a potential probiotic because it can produce short-chain fatty acids (Quevrain et al., 2016; Lenoir et al., 2020). Therefore, in the present study, we conducted annotations of all the proteins of *F. prausnitzii* strains and paid more attention to the metabolic pathways of fatty acids. The pathway of fatty acid metabolism was constructed to provide more valuable information about the beneficial functions of *F. prausnitzii* strains. EnrichM (Liu et al., 2021) was used to classify and obtain the KO IDs of the *F. prausnitzii* strains. These KO IDs were then used to construct the fatty acid metabolism pathway *via* KEGG Mapper – Reconstruct Pathway (Kanehisa et al., 2017) (**Figure 3C**). As shown in **Figure 3C**; **Supplementary Figure 3**, and **Supplementary Table 9**, the complete metabolic pathway of *F. prausnitzii* strains consists of different enzymes that transform different substrates. The initial substrate of this process is acetyl-CoA, which undergoes the addition of a molecule of malonyl-[acp] and sequentially reduces, oxidizes, and reduces to eliminate carbonyl, thus introducing two carbon atoms to synthesize butanoyl-[acp] containing four carbon atoms. The introduction of two carbon atoms occurs every time these four steps of the reaction are carried out until stearoyl-CoA/ACP with 18 carbon atoms is synthesized (Kindt et al., 2018).

All enzymes involved in fatty acid metabolism and their expression levels in each strain of *F. prausnitzii* are summarized in **Supplementary Tables 9**, **10**. K00208 (FabI), K01961 (accC), and K16363 (IpxC-FabZ) were not used to construct the pathway because they were only found in *F. prausnitzii* BIOML-B16 and *F. prausnitzii* 2789STDY5834930 (**Supplementary Table 10**). Other enzymes with the same functions as these three enzymes can be found in other *F. prausnitzii* strains. For instance, K00208 (FabI) and K02371 (FabK) are both enoyl-[acyl-carrier protein] reductases. Therefore, the pathway constructed after discarding these three enzymes can more reasonably show the fatty acid metabolism of *F. prausnitzii*.

Several previous studies have demonstrated the great potential of *F. prausnitzii* strains for treating intestinal diseases due to the ability of their fatty acid metabolism (Quevrain et al., 2016; Lenoir et al., 2020). Overall, these results provide an in-depth understanding of *F. prausnitzii* strains and suggest diverse functions, in particular with regard to their abundant BGCs and their fatty acid metabolic pathway, which strongly prove their potential in the treatment of diseases related to gut microbiota.

### CRISPR/Cas system components in *F. prausnitzii* strains

To obtain an in-depth understanding of the genome and efficiently edit the genetic elements of *F. prausnitzii* strains, we detected the components of the CRISPR/Cas systems, including the Cas proteins and the CRISPR arrays, for all 84 *F . prausnitzii* strains using the online tool CRISPRCasFinder (the results are summarized in **Supplementary Table 11**). Specifically, four types of Cas proteins with clear classifications, including CAS-Type IE, CAS-Type IC, CAS-Type IIIA, and CAS-Type ID, and one predicted Cas protein (CAS_putative), were identified in most *F. prausnitzii* strains, except *F. prausnitzii* A2-165, *F. prausnitzii* CNCM | 4540, *F. prausnitzii* A2165, *F. prausnitzii* APC942/8-14-2, *F. prausnitzii* AM36-18BH, *F. prausnitzii* JCM 31915, *F. prausnitzii* MGYG-HGUT-00022, *F. prausnitzii* MGYG-HGUT-02272, and *F. prausnitzii* 2789STDY5834930 (**Supplementary Table 11**). After checking the sizes and assembly levels of the genomes for these strains, we speculated that the presence of incomplete genomes affects the identification results. Of these five kinds of Cas proteins, CAS_putative was identified in most *F. prausnitzii* strains, while most *F. prausnitzii* strains (52.3%) contained CAS-Type IE, and only four strains contained CAS-Type ID. In addition, the number of Cas proteins in the 84 *F . prausnitzii* strains ranged from one to seven (**Supplementary Table 11**). The distribution of Cas proteins in the 84 *F . prausnitzii* strains may be associated with extensive horizontal gene transfer and rapid evolution (Soucy et al., 2015), suggesting a large variation of the CRISPR/Cas system among the strains.

In addition, CRISPR arrays of the 84 *F . prausnitzii* strains were also identified, which can indicate their ability to accept foreign DNA. Our results showed that the number of CRISPR arrays in *F. prausnitzii* varied. For example, *F. prausnitzii* FPSSTS7063_SV_a2_mod has the largest number of CRISPR arrays (nine). By contrast, fifteen strains, namely, *F. prausnitzii* APC923/51-1, *F. prausnitzii* AM37-13AC, *F. prausnitzii* AM36-18BH, *F. prausnitzii* 2789STDY5834930, *F. prausnitzii* CNCM | 4546, *F. prausnitzii* BIOML-B7, *F. prausnitzii* BIOML-B8, *F. prausnitzii* BIOML-B9, *F. prausnitzii* BIOML-B10, *F. prausnitzii* BIOML-B11, *F. prausnitzii* BIOML-B12, *F. prausnitzii* BIOML-B13, *F. prausnitzii* BIOML-B14, *F. prausnitzii* BIOML-B15, and *F. prausnitzii* BIOML-B16 have only one CRISPR array (**Supplementary Table 11**). These results provide a more in-depth understanding of the genetic elements of *F. prausnitzii* and its potential clinical applications.

### Evaluation of the potential risk of *F. prausnitzii* strains based on the profiles of ARGs, VFs, and PGs

**Figure 5:**
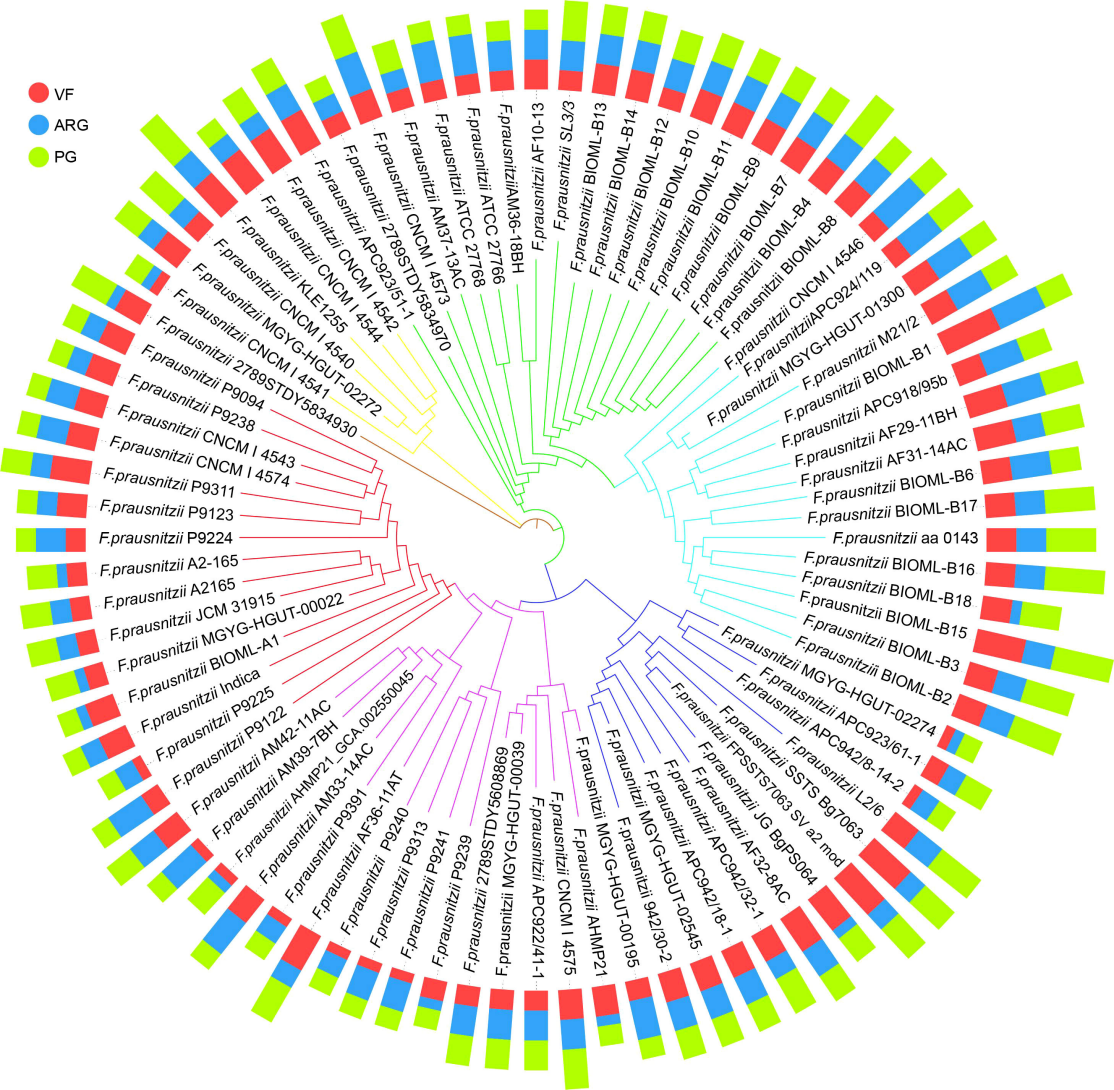
The profiles of VFs, ARGs, and PGs in 84 F*. prausnitzii* strains. Based on the sequences of 303 single-copy proteins, we constructed the phylogenetic tree (the inner circle) using the neighbor joining method. The outer circle is the bar graph of the three risk factors VF (red), ARG (blue), and PG (green). The types of risk factors are shown in **Supplementary Tables 12**, **14**, and **16**. The specific number of each risk factor carried by each strain is shown in **Supplementary Tables 13**, **15**, and **17**.

In recent years, the safety problems and adverse effects caused by using probiotics have been considered (Marissen et al., 2019; Rittiphairoj et al., 2019). Moreover, previous studies have pointed out that *F. prausnitzii* can be used for microbiological therapies to treat several intestinal diseases, such as IBS and CD (Quevrain et al., 2016). Therefore, when considering *F. prausnitzii* as a potential next-generation probiotic, it is essential to determine the potential risk of these *F. prausnitzii* strains to guide the selection of high-security strains. To solve this issue, we profiled the composition of ARGs, VFs, and PGs. Specifically, eight types of ARGs were identified in *F. prausnitzii* strains, namely, *aad*E, *erm*C, *tet*40, *tet*W, *cat*D, *tet*A, *erm*B, and *aph* (3’)-I (**Figure 5**; **Supplementary Tables 12**, **13**), suggesting that the *F. prausnitzii* strains possess resistance to aminoglycosides, macrolide-lincosamide-streptogramin, tetracycline, and chloramphenicol. Similarly, eight types of VFs were detected in the *F. prausnitzii* strains, ranging from one to six per strain (**Figure 5**; **Supplementary Tables 14**, **15**). Eight types of VFs were detected in *F. prausnitzii* strains, namely, putative galactosyltransferase, polysaccharide biosynthesis protein_CpsF, capsular polysaccharide biosynthesis protein Cps4J, elongation factor Tu, chaperonin GroEL, ATP-dependent Clp protease proteolytic subunit, glucose-1-phosphate thymidylyltransferase, and UDP-N-acetylglucosamine 2-epimerase (**Figure 5**; **Supplementary Table 14**). In addition, 12 types of PGs were identified in *F. prausnitzii* strains: *Tuf*A, *Cps*2J, *Clp*P, *Clp*X, *Pst*B, *Rml*A, *Rec*A, *Oad*B, *Cps2*E, *Arg*G, *Msa*B, and *HpHSP*60 (**Figure 5**; **Supplementary Tables 16**, **17**). The distributions of ARGs, VFs, and PGs in the 84 *F . prausnitzii* strains were diverse, which revealed that the potential risks of *F. prausnitzii* strains are varied and that these factors can be used to reflect the risk of each strain.

**Figure 6:**
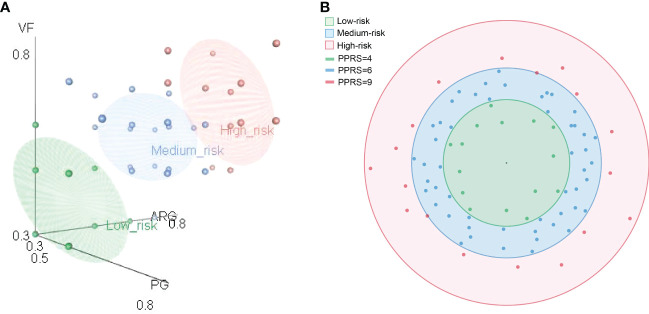
Comprehensive probiotic potential risk model of *F. prausnitzii*. **(A)** Eighty-four *F. prausnitzii* strains were classified as either low risk (green), medium risk (blue), or high risk (red) according to their probiotic potential risk index (PPRI). **(B)** Eighty-four *F. prausnitzii* strains were classified as either low risk (green), medium risk (blue), or high risk (red) according their probiotic potential risk score (PPRS).

Therefore, based on the composition of ARGs, VFs, and PGs, we proposed two indices, namely, the PPRI and the PPRS. To show the composition of risk factors, PPRI was designed as a one-dimensional combination vector with three variables. To make PPRI more intuitive, we designed a PPRS that calculated the Euclidean distance of *F. prausnitzii* strains based on their PPRI. The PPRI and PPRS of each *F. prausnitzii* strain are summarized in **Supplementary Table 18** and visualized in **Figure 6**. As shown in **Figure 6A**, the three coordinate axes represent the PPRI of the three indicators, ARGs, VFs, and PGs, for each *F. prausnitzii* strain. Subsequently, we calculated the PPRS of each *F. prausnitzii* strain (visualized in **Figure 6B**). The PPRS values ranged from 2.5 to 8.4 (**Supplementary Table 18**). Combining these two indices, we classified these 84 *F . prausnitzii* strains into three groups, namely, low-risk (green, PPRS ≤4), medium-risk (yellow, 4< PPRS<6), and high-risk groups (red, PPRS ≥6).

Finally, *F. prausnitzii* CNCM | 4541, *F. prausnitzii* MGYG-HGUT-02274, *F. prausnitzii* APC942/8-14-2, *F. prausnitzii* P9391, *F. prausnitzii* P9225, *F. prausnitzii* P9239, *F. prausnitzii* P9240, *F. prausnitzii* AHMP21_GCA.002550045, *F. prausnitzii* 2789STDY5834970, *F. prausnitzii* BIOML-A1, *F. prausnitzii* A2-165, *F. prausnitzii* AHMP21, *F. prausnitzii* P9241, *F. prausnitzii* P9313, and *F. prausnitzii* MGYG-HGUT-00022 were classified into the low-risk group (**Supplementary Table 18**). These results suggest that these 15 bacteria can be selected as potential candidate strains for use in clinical treatment.

These two indices, PPRI and PPRS, were first proposed in the present study. They were designed not only for *F. prausnitzii* strains but also for other probiotics. These two indices are only indicators for risk assessment based on the genetic features of the strains. It should be noted that three features, including ARGs, VFs, and PGs, were selected for the construction of the PPRI and PPRS. If necessary, more multiple features, such as phenotypic data and wet experimental data, can be used as supplemental features to implement the PPRI and PPRS approaches. Of course, our assessment method can only provide guidance for the selection of probiotics, including *F. prausnitzii* strains.

## Conclusion

In the present study, to obtain a comprehensive understanding of the genetic diversity, functional characteristics, and potential risks of *F. prausnitzii*, we collected the available genetic information of 84 *F . prausnitzii* strains to conduct a pan-genome analysis. We generated 8,420 and 375 homologous clusters as the pan-genome and core genome for *F. prausnitzii*, respectively. Phylogenetic analysis of the 84 strains revealed that although these strains can be divided into six groups, there were great discrepancies in the composition of the phylogenetic trees constructed by three different strategies, which indicated that the compositions of genetic elements of the 84 *F . prausnitzii* strains were varied. Based on the pan-genome of *F. prausnitzii*, we profiled the composition of functional traits against the COG, CAZyme, KEGG, ARG, VF, and PHI databases, predicted the BGCs, and constructed the fatty acid metabolism pathway for *F. prausnitzii* strains. Our results showed that the proteins of the accessory group made a greater contribution to the primary genetic functions of the *F. prausnitzii* strains than those of the core and specific groups, based on the COG annotations. Only a small number of proteins related to human diseases were identified according to the KEGG annotations, and no secondary metabolic gene clusters encoding harmful products were detected in the *F. prausnitzii* strains. In particular, the construction of a complete fatty acid metabolism pathway showed the metabolic ability of the synthesis of fatty acids. To obtain an in-depth understanding of the genome and efficiently edit the genetic elements of *F. prausnitzii* strains, the components of the CRISPR/Cas system were identified. Four types of Cas proteins with a clear classification and one predicted Cas protein were identified in most *F. prausnitzii* strains, and our results showed that the number of CRISPR arrays in *F. prausnitzii* varied. To reveal the potential risks of *F. prausnitzii* strains, we detected harmful elements; eight types of ARGs, eight types of VFs, and 12 types of PGs were detected. To evaluate the potential risks, we first proposed the PPRI and PPRS to classify these 84 strains into low-, medium-, and high-risk groups and identified 15 strains as low-risk strains that should be prioritized for clinical application. Overall, the present study provides a comprehensive understanding of the genetic diversity, functional characteristics, and metabolic pathways of *F. prausnitzii*. In particular, we propose the PPRI and PPRS for use in evaluating the potential risks of probiotics in general (not just *F. prausnitzii*) and guiding their clinical treatment. Our results provide insight into the understanding and application of *F. prausnitzii* as a next-generation probiotic.

## Data availability statement

The original contributions presented in the study are included in the article/**Supplementary Material**. Further inquiries can be directed to the corresponding authors.

## Author contributions

GL and MH designed the study. NZ, YJ, FF, and YL collected the data. ZB and MH conducted the pan-genome analysis. ZB, NZ, LC, and MH drew the figures. ZB, MH, and GL wrote the initial draft of the manuscript. All authors read, modified, and approved the final manuscript.
