# Supplementary material for: Comprehensive analysis of 84 Faecalibacterium prausnitzii strains uncovers their genetic diversity, functional characteristics, and potential risks
Source: Front Cell Infect Microbiol. 2023 Jan 4;12:919701. doi: 10.3389/fcimb.2022.919701 (PMC9846645; doi:10.3389/fcimb.2022.919701)
Supplement: Supplementary file 1 [file DataSheet_1.docx]

**Comprehensive analysis of 84 *Faecalibacterium prausnitzii* strains uncovers their genetic diversity, functional characteristics, and potential risks**

Zipeng Bai^1, †^, Na Zhang^1, †^, Yu Jin^2^, Long Chen^1^, Yujie Mao^1^, Lingna Sun^1^, Feifei Fang^2^, Ying Liu^2^, Maozhen Han^1,*^, Gangping Li^2,*^

1 School of Life Sciences, Anhui Medical University, Hefei, Anhui 230032, China.

2 Division of Gastroenterology, Union Hospital, Tongji Medical College, Huazhong University of Science and Technology, Wuhan, 430022, China.

† These authors contributed equally to this work.

*Corresponding authors: ligangping@hust.edu.cn; [hanmz@ahmu.edu.cn](mailto:hanmz@ahmu.edu.cn)


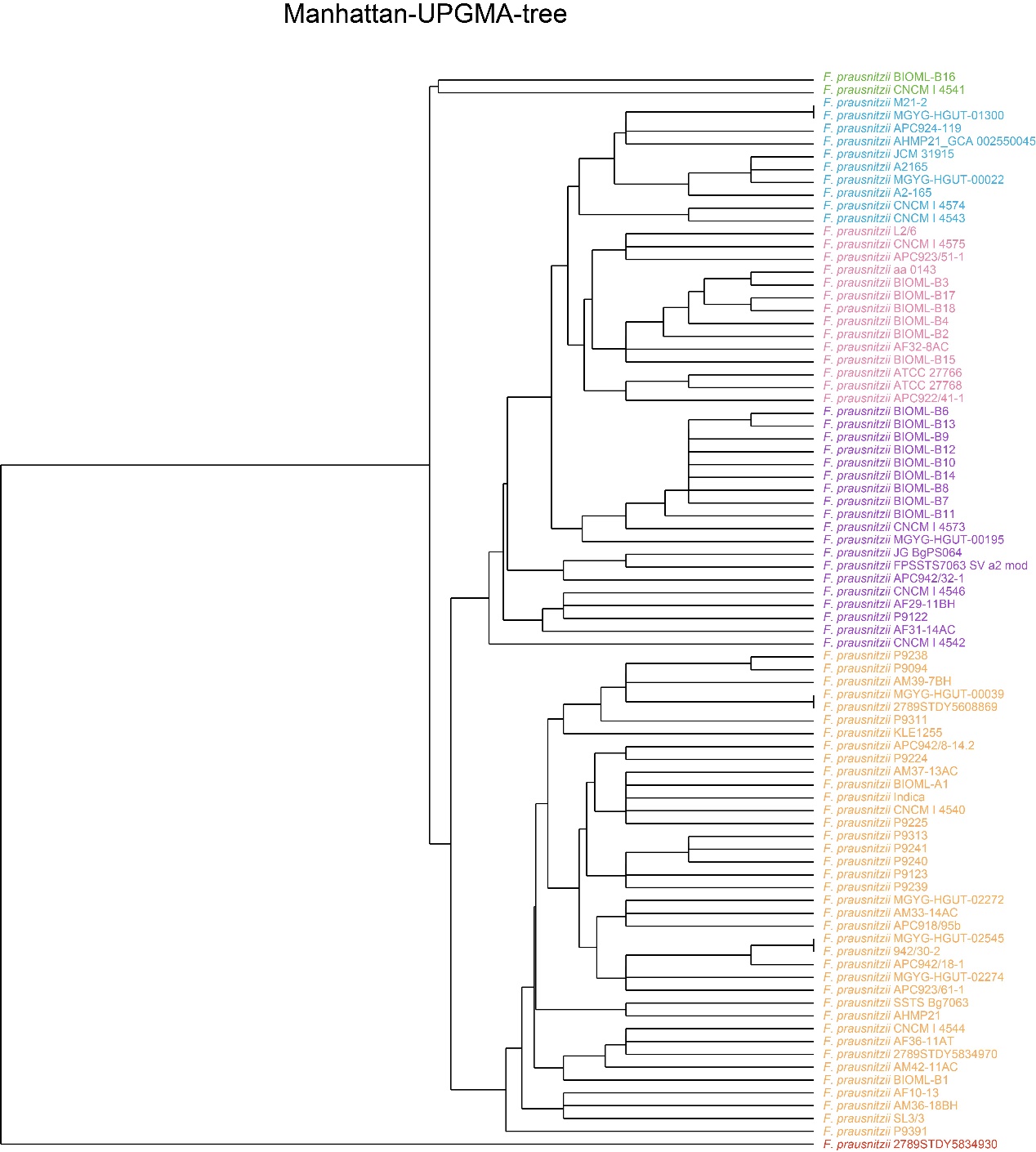


**Supplementary Figure 1: UPGMA tree of *F. prausnitzii*.** Phylogenetic tree was constructed with the method of unweighted group average method (UPGMA). These 84 *F. prausnitzii* strains can be divided into six groups according to their evolutionary relationship.


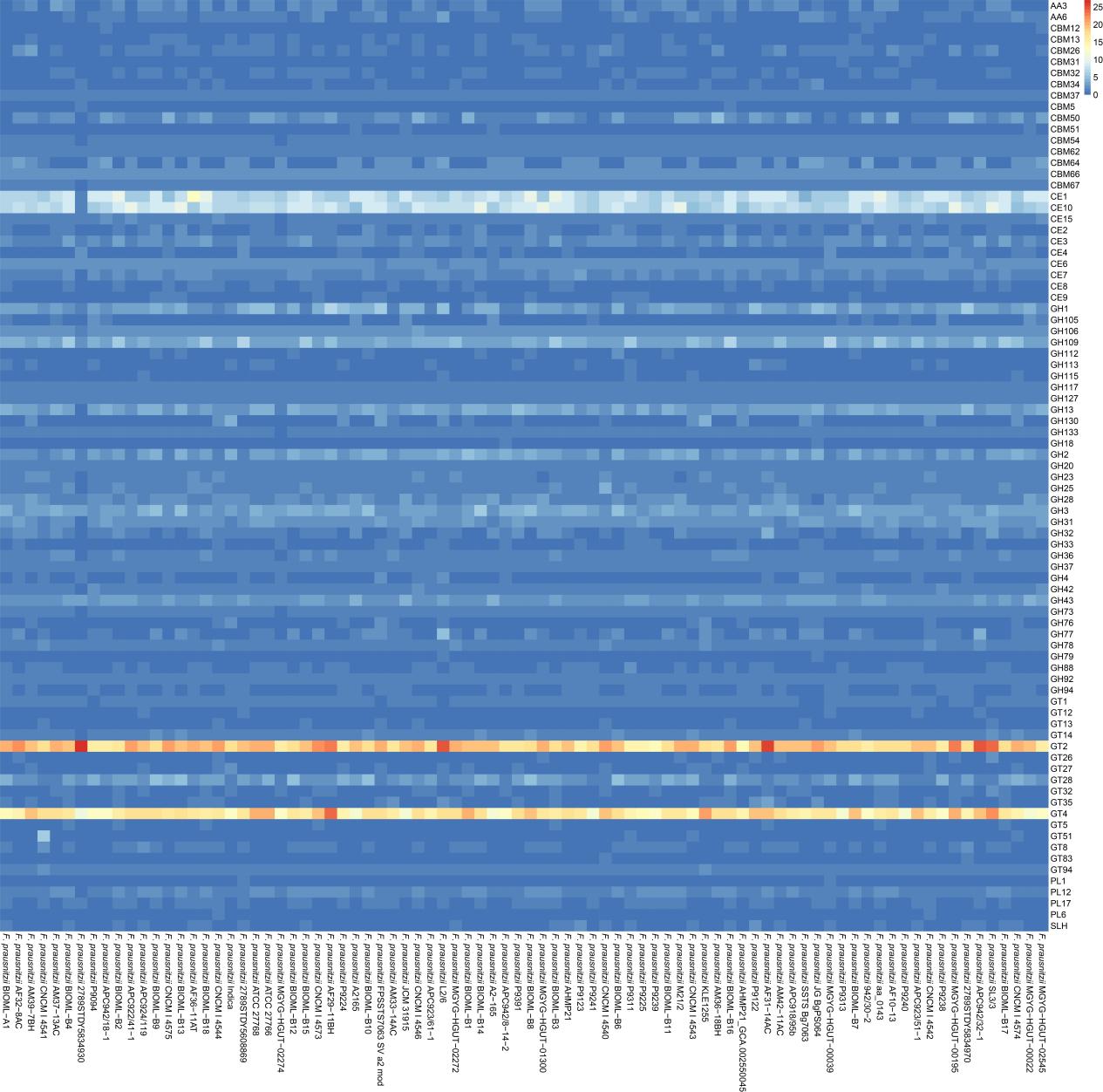


**Supplementary Figure 2: Detailed results of the CAzyme database annotations.** The number of each carbohydrate active enzyme annotated in the 84 *F. prausnitzii* strains are shown. It can be seen that there are a large number of Glycoside Transferases (GTs) and Carbohydrate Esterases (CEs) carried by *F. prausnitzii*, especially GT2, GT4, CE1, and CE10.


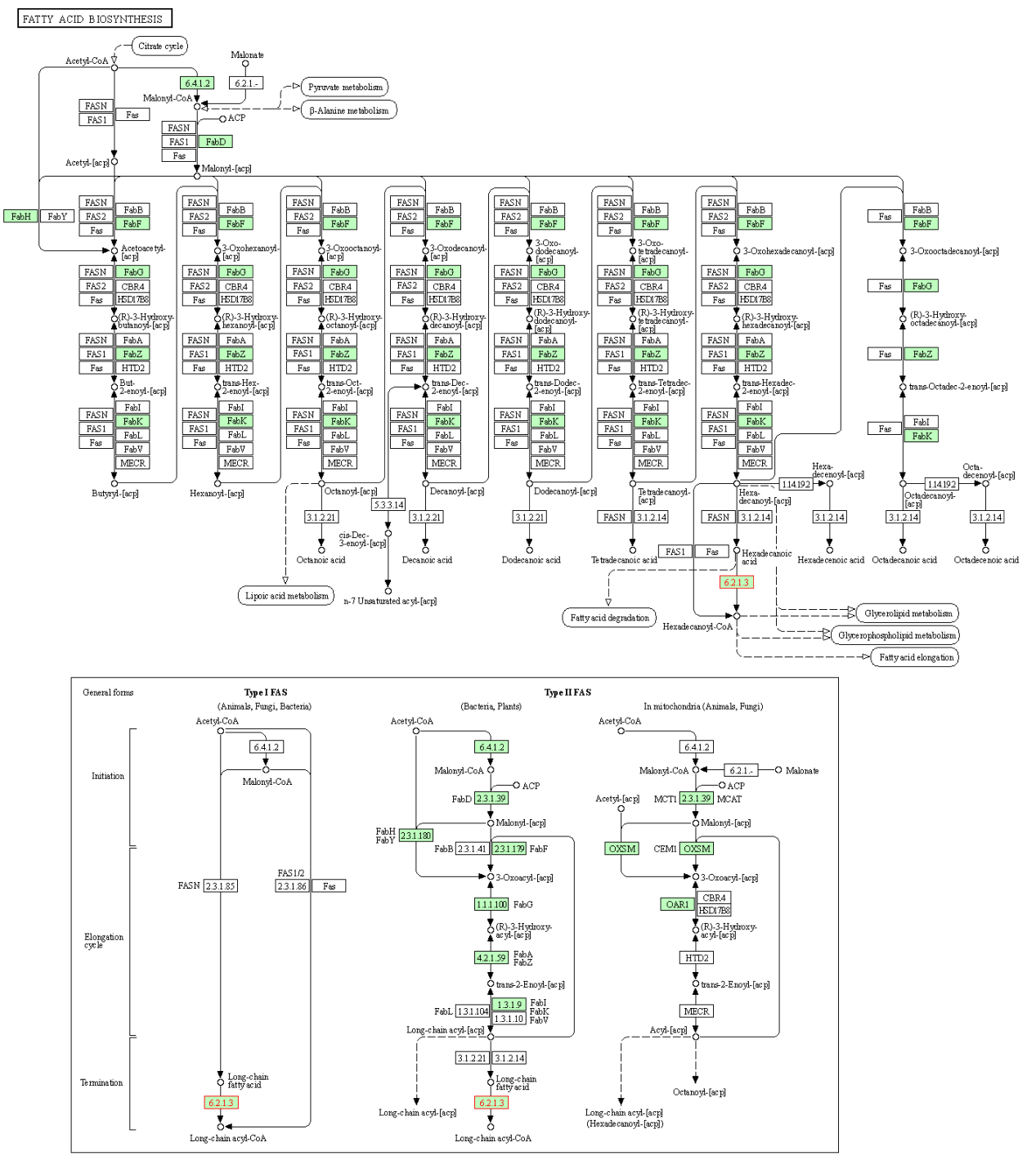


**Supplementary Figure 3: Detailed results of pathway of fatty acid metabolism of *F. prausnitzii* constructed by KEGG.** Each enzyme associated with fatty acid metabolism in the 84 *F. prausnitzii* strains is labeled. It can be seen that *F. prausnitzii* has a complete ability to metabolize fatty acids.
